# JA signal-mediated immunity of *Dendrobium catenatum* to necrotrophic Southern Blight pathogen

**DOI:** 10.1186/s12870-021-03134-y

**Published:** 2021-08-06

**Authors:** Cong Li, Qiuyi Shen, Xiang Cai, Danni Lai, Lingshang Wu, Zhigang Han, Tianlun Zhao, Donghong Chen, Jinping Si

**Affiliations:** 1 State Key Laboratory of Subtropical Silviculture, Zhejiang A&F University, Hangzhou, 311300 China; 2 College of Agriculture and Biotechnology, Zhejiang University, Hangzhou, 310058 China

**Keywords:** *Dendrobium catenatum*, Jasmonate, *Sclerotium delphinii*, COI1, JAZ, MYC transcription factor

## Abstract

**Background:**

*Dendrobium catenatum* belongs to the Orchidaceae, and is a precious Chinese herbal medicine. In the past 20 years, *D. catenatum* industry has developed from an endangered medicinal plant to multi-billion dollar grade industry. The necrotrophic pathogen *Sclerotium delphinii* has a devastating effection on over 500 plant species, especially resulting in widespread infection and severe yield loss in the process of large-scale cultivation of *D. catenatum*. It has been widely reported that Jasmonate (JA) is involved in plant immunity to pathogens, but the mechanisms of JA-induced plant resistance to *S. delphinii* are unclear.

**Results:**

In the present study, the role of JA in enhancing *D. catenatum* resistance to *S. delphinii* was investigated. We identified 2 COI1, 13 JAZ, and 12 MYC proteins in *D. catenatum* genome. Subsequently, systematic analyses containing phylogenetic relationship, gene structure, protein domain, and motif architecture of core JA pathway proteins were conducted in *D. catenatum* and the newly characterized homologs from its closely related orchid species *Phalaenopsis equestris* and *Apostasia shenzhenica*, along with the well-investigated homologs from *Arabidopsis thaliana* and *Oryza sativa*. Public RNA-seq data were investigated to analyze the expression patterns of *D. catenatum* core JA pathway genes in various tissues and organs. Transcriptome analysis of MeJA and *S. delphinii* treatment showed exogenous MeJA changed most of the expression of the above genes, and several key members, including *DcJAZ1/2/5* and *DcMYC2b*, are involved in enhancing defense ability to *S. delphinii* in *D. catenatum*.

**Conclusions:**

The findings indicate exogenous MeJA treatment affects the expression level of *DcJAZ1/2/5* and *DcMYC2b*, thereby enhancing *D. catenatum* resistance to *S. delphinii*. This research would be helpful for future functional identification of core JA pathway genes involved in breeding for disease resistance in *D. catenatum*.

**Supplementary Information:**

The online version contains supplementary material available at 10.1186/s12870-021-03134-y.

## Background

*Dendrobium* of Orchidaceae is an importantly economical genera, which comprises nearly 1,600 accepted species [1, 2]. Among them, *D. catenatum* (also known as *D. officinale*) is a popular tonic and traditional Chinese medicine herb with prominent commercial value, including protection against upset stomach, promotion of body fluid production, immune enhancement, nourishing Yin, as well as antioxidant and antitumor effects [3]. In the past 20 years, scientists have successfully overcome the bottleneck problems against *D. catenatum* industrious development, such as seedling breeding and facility cultivation [3], which makes it from an endangered medicinal plant to multi-billion dollar grade industry [4]. However, in the process of large-scale cultivation of *D. catenatum*, Southern Blight disease caused by *Sclerotium delphinii* leads to heavy yield losses by infecting its leaves, stems, and roots [5].

Southern Blight is a broad-spectrum disease caused by the necrotrophic pathogen *S. delphinii* and *S. rolfsii* [5, 6]. *Athelia rolfsii* is the sexual state of the fungus, we rarely see it, while its asexual state *S. delphinii* and *S. rolfsii* are more common [6]. Compared with *S. rolfsii*, *S. delphinii* is more pathogenic [5]. In warm and high humidity environments, *S. delphinii* infects the host and secretes many cell wall-degrading enzymes (CWDEs) to degrade the host cell wall [7], and then hyphae penetrate the plant tissue causing local necrosis, with subsequent formation of abundance of white mycelium and rapeseed-like sclerotia [5]. Southern Blight is considered one of the most destructive diseases worldwide for its vast host range of over 500 plant species, including various crops, vegetables, fruits, ornamental flowers, medicinal plants, etc., resulting in an average economic loss of 25% [8], and even 80% in severe cases [7]. Furthermore, its sclerotia can survive in soil for over five years even in the absence of hosts [7]. At present, biological control agents including *Rhizobium* [9], *Trichoderma* [10], and *Streptomyces* [7], and chemical control fungicides like tebuconazole and flutolanil [11], have been reported. However, biocontrol has the problem of inefficiency and fungicide utilization leads to bio-magnification in the food chain and environmental pollution [9]. Therefore, new control strategy is urgently needed.

The lipid-derived hormones, jasmonates (JAs), regulate plants defense against necrotrophic pathogens and a wide variety of herbivores [12–14]. Necrotrophic pathogens affected by JA-induced plant defenses include the bacterial pathogens *Xanthomonas oryzae* and *Pectobacterium atrosepticum* [15], fungal pathogens such as *Alternaria brassicicola*, *Botrytis cinerea*, *Sclerotinia sclerotiorum*, *Plectosphaerella cucumerina*, *Fusarium oxysporum*, and *oomycete Pythium spp* [12, 13]. The bioactive form jasmonoyl-L-isoleucine (JA-Ile) is produced by conjugation of jasmonic acid with isoleucine in the plant cytosol [16], which is perceived through a nuclear co-receptor composing of CORONATINE INSENSITIVE1 (COI1), the F-box subunit of the ubiquitin ligase SCF^COI1^ [17], and JASMONATE ZIM-DOMAIN (JAZ) proteins [18–21]. In *Arabidopsis,* the *COI1* gene encodes a 66-kD protein with 16 leu-rich repeats (LRRs) and an F-box motif [22]. In ‘stress-free’ conditions with low levels of JA-Ile, JA responses are repressed by a group of JAZ proteins [16, 19–21]. JAZs belong to the TIFY super-family that is defined by a highly conserved TIFY motif (TIF[F/Y] XG) residing within the ZIM domain [19–21, 23]. In *Arabidopsis*, 13 genes (*JAZ1*-*JAZ13*) encode JAZ proteins with a highly conserved Jas motif (also known as a CCT_2 motif) at the C terminus [24, 25]. JAZ proteins have a strong affinity on some related bHLH TFs such as MYC2, MYC3, MYC4, and MYC5 as well as GL3 (GLA BRA3), EGL1 (ENHANCE R OF GLA BRA3 1), and TT8 (TRANSPARENT TE STA 8), involved in both specific and overlapped pathways of JA [26, 27]. These bHLH-type MYC TFs share highly sequence homology both in bHLH and bHLH-MYC_N (containing JAZ-interacting domain (JID) domain) domains [27, 28]. Of these JAZ targeted TFs, MYC2 is best-characterized and considered as a regulatory hub of the JA signaling pathway [29–31]. JAZ repressors physically bind and inhibit MYCs [20, 24] through two distinct mechanisms [19–21]. First, MYC-bound JAZ proteins recruit the co-repressors TOPLESS (TPL) either directly by ETHYLENE-RESPONSE FACTOR Amphiphilic Repression (EAR) motifs located at the N terminus of a subset of JAZ proteins (e.g., JAZ7, JAZ8, JAZ13) or indirectly through NOVEL INTERACTOR OF JAZ (NINJA), an EAR motif-containing protein [32–34]. Second, the Jas motif of JAZ binding to the N-terminal JID of MYCs blocks access of MYCs to the MED25 coactivator subunit of the mediator complex [35–37]. Several JAZ repressors belong to AtJAZI and AtJAZIII clade also contain an N-terminal cryptic MYC-interaction domain (CMID), which binds MYCs more tightly than the Jas motif [26, 38–42]. In response to stress cues, JA-Ile was synthesized, which directly promotes the interaction between COI1 and JAZ, and subsequent SCF^COI1^-dependent degradation of JAZ repressors occurred [18, 43]. The liberation of MYCs from repressive state results in extensive transcriptional reprograming and a plethora of JA-dependent physiological responses [44, 45]. Hence, it is significant to exploit the role of JA pathway genes against Southern Blight in *D. catenatum* molecular breeding.

Here, we employed a genetic approach to identify the members of COI, JAZ, and MYC family of *D. catenatum*, respectively, and subsequently assessed the phylogenetic relationship, gene structure, and domain architecture, and then showed exogenous MeJA enhanced *D. catenatum* resistance to *S. delphinii* through phenotype identification and gene expression profiling in the core JA pathway. The results provide the basis for further functional characterization of *DcCOIs*, *DcJAZs*, and *DcMYCs*, and in particular, their role in *D. catenatum* response to *S. delphinii*.

## Results

### Exogenous jasmonates enhanced D. catenatum resistance to S. delphinii

**Fig. 1:**
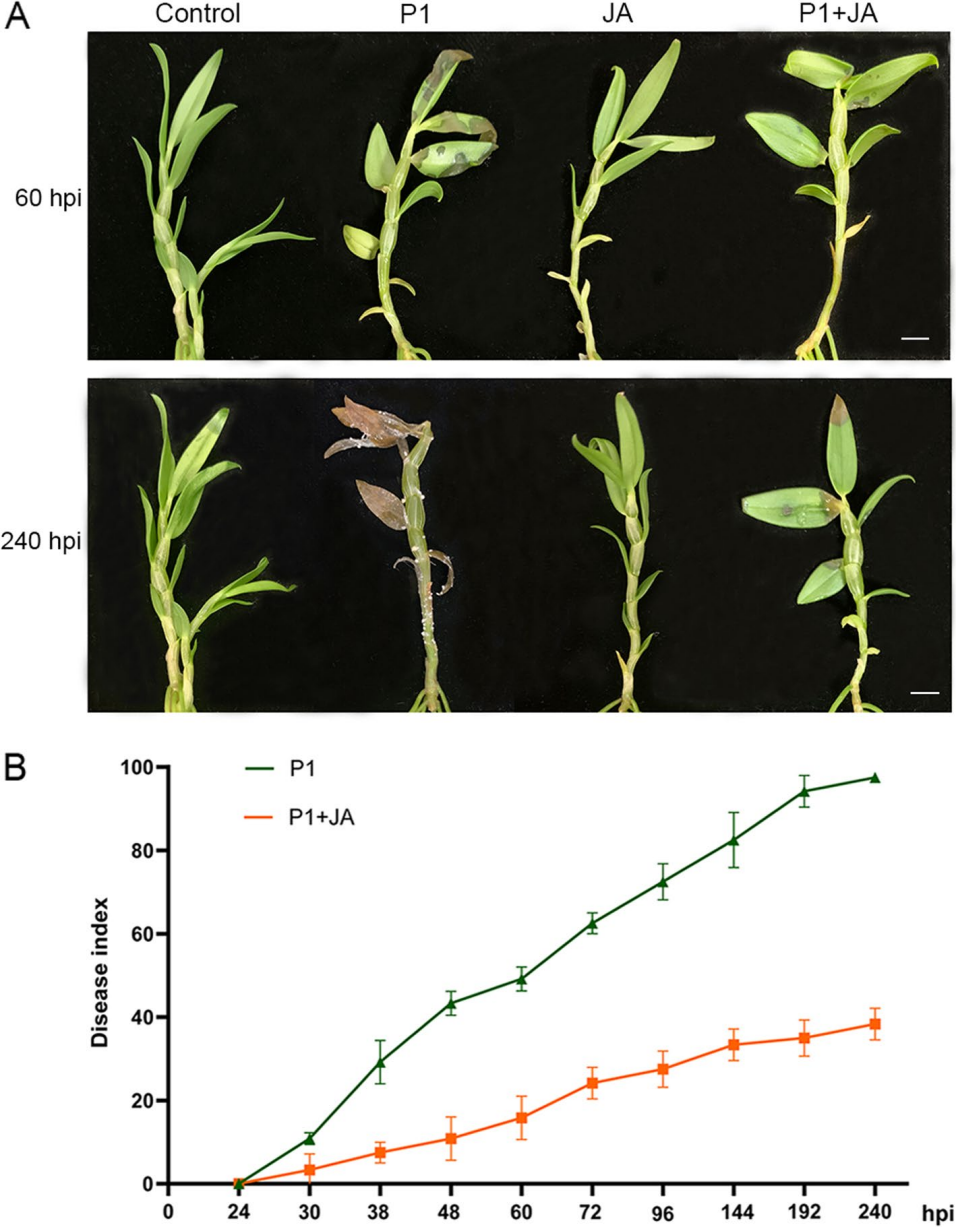
Effect of exogenous MeJA pre-treatment on *D. catenatum* resistance to *S. delphinii*. **A** Disease symptoms of *D. catenatum* plantlets pre-treated with 1 mM MeJA following inoculation with *S. delphinii* at 60 and 240 hpi. Bar = 1 cm. **B** Disease indexes of the MeJA pre-treated *D. catenatum* were determined from 24 hpi (1 dpi) to 240 hpi (10 dpi). The values are the means ± SD; *n* = 10

Pre-treated with 1 mM MeJA for 4 h, four-month-old *D. catenatum* plantlets were inoculated with *S. delphinii* mycelia suspensions. Different responses to the disease infection were observed in control and MeJA-pretreated plantlets (Fig. 1). The symptoms, water-stained and brown necrotic lesions, were much more severe in the control than in pre-treated plantlets at 60 hpi (Fig. 1A). With prolonged hpi, the disease became more and more serious, but disease development was much slower in the pre-treated plantlets compared with the control. At 144 hpi (6 dpi), the disease index was 82.50 and 33.33% for the control and MeJA pre-treated plantlets, respectively (Fig. 1B). While almost all control plantlets were nearly dead and covered with white sclerotia at 240 hpi (10 dpi), pre-treated plantlets showed less necrotic lesions and no visible mycelia or sclerotia (Fig. 1A).

## Identification of D. catenatum core JA signaling pathway genes

In view of the above-stated phenotypic results, we screened and identified the JA pathway genes in *D. catenatum*. The COI protein sequences of *Arabidopsis* and rice were used as queries to BLASTP search against the *D. catenatum* and its close relative *P. equestris* and *A. shenzhenica* datasets. In total, two COI candidate protein sequences were obtained in the *D. catenatum* genome sequences, while only one was identified in the other two orchid species, respectively, suggesting that specific duplication of COI family genes happened in *D. catenatum* (Tables 1, S1, and S2). To understand the role of gene functions, subcellular localization prediction for *DcCOI*, *PeCOI* and *AsCOI* genes using WoLE PSORT were performed. It was showed that *DcCOI* and *PeCOI* genes exhibited cytoplasmic localization and *AsCOI* exhibited chloroplast localization. Moreover, the MW and pI of the COI proteins were calculated using the Protparam tool in ExPASy. The MW of COIs varied from 66,194.57 to 76,380.33 Da, and the pIs ranged from 6.31 to 8.11. The length, MW, pI of DcCOIs and PeCOI proteins were similar, and lower than that of AsCOI, suggesting that COI protein was more conservative in epiphytic orchid.

**Table 1:** Characteristics of core JA signaling pathway genes in *D. catenatum*

Gene Family	No	Name	Synonym	Gene ID	Clade	Exon Number	Protein(aa)	Isoform	pI	MW(Da)	Localization Predicted
COI Family	1	DcCOI1a		LOC110108433		3	587		6.55	66,596.93	Cytoplasmic
	2	DcCOI1b		LOC110114189		3	586		6.68	66,194.57	Cytoplasmic
TIFY Family	1	DcTIFY1	DcZML1	LOC110094350	ZML	8	279	0	6.74	30,138.16	Nuclear
	2	DcTIFY2a	DcZML3	LOC110110163	ZML	7	261	0	8.26	28,094.56	Nuclear
	3	DcTIFY2b	DcZML2	LOC110111293	ZML	8	352	0	6.79	38,623.68	Nuclear
	4	DcTIFY3	DcJAZ6	LOC110103561	JAZII	5	216	2	5.5	22,533.06	Plasma membrane
	5	DcTIFY4	DcPPD	LOC110092376	PPD	9	357	2	9.51	38,991.08	Nuclear
	6	DcTIFY5a	DcJAZ9	LOC110112116	JAZIV	3	155	0	8.91	16,572.53	Nuclear
	7	DcTIFY5b	DcJAZ10	LOC110104985	JAZIV	3	142	0	8.4	15,482.58	Nuclear
	8	DcTIFY5c	DcJAZ11	LOC110110139	JAZIV	3	147	0	7.73	16,502.96	Nuclear
	9	DcTIFY5d	DcJAZ12	LOC110103420	JAZIV	3	156	0	6.37	17,481.81	Nuclear
	10	DcTIFY5e	DcJAZ13	LOC110097296	JAZIV	1	123	0	9.34	13,912.86	Nuclear
	11	DcTIFY6a	DcJAZ7	LOC110094727	JAZV	7	380	0	8.7	40,866.66	Nuclear
	12	DcTIFY6b	DcJAZ8	LOC110103933	JAZV	7	401	2	9.29	43,134.7	Nuclear
	13	DcTIFY8	DcTIFY8	LOC110096617	TIFY	6	414	0	7.13	43,803.04	Nuclear
	14	DcTIFY10a	DcJAZ1	LOC110108135	JAZI	5	239	0	8.55	25,603.29	Chloroplast
	15	DcTIFY10b	DcJAZ2	LOC110113635	JAZI	5	250	0	8.43	27,286.8	Chloroplast
	16	DcTIFY10c	DcJAZ3	LOC110116626	JAZI	5	268	0	9.18	29,731.97	Nuclear
	17	DcTIFY10d	DcJAZ4	LOC110104382	JAZI	5	235	2	8.53	26,416.69	Nuclear
	18	DcTIFY10e	DcJAZ5	LOC110107212	JAZI	5	217	2	6	24,268.43	Nuclear
MYC Family	1	DcMYC2a		LOC110116479	MYCI	1	651	0	5.53	74,540.42	Nuclear
	2	DcMYC2b		LOC110114462	MYCI	1	636	0	6.88	42,301.88	Nuclear
	3	DcMYC2c		LOC110092865	MYCI	1	656	0	5.4	73,818.02	Nuclear
	4	DcMYC2d		LOC110094435	MYCI	1	462	0	5.12	74,172.35	Nuclear
	5	DcJAM1a		LOC110094094	MYCII	1	499	0	5.89	45,777.65	Nuclear
	6	DcJAM1b		LOC110103241	MYCII	3	558	0	5.98	45,803.81	Nuclear
	7	DcJAM4a		LOC110113808	MYCII	1	381	0	6.92	61,580.84	Nuclear
	8	DcJAM4b		LOC110099101	MYCII	1	419	0	5.83	55,244.9	Nuclear
	9	DcJAM4c		LOC110116682	MYCII	1	422	0	5.7	69,408.72	Nuclear
	10	DcTT8		LOC110097687	MYCIII	9	662	0	5.18	51,218.00	Nuclear
	11	DcEGL3		LOC110114654	MYCIV	9	653	0	5.24	72,046.99	Nuclear
	12	DcGL3		LOC110111891	MYCIV	10	644	0	5.95	72,002.67	Nuclear

A genome-wide search of TIFY family genes in *D. catenatum*, *P. equestris*, and *A. shenzhenica* datasets yielded 18, 20, and 13 non-redundant genes that were found to harbor the TIFY domain (Tables 1, S1, and S2). Of these TIFY domain-containing genes, 13, 14, and 7 contained both the TIFY domain and the Jas motif (PF09425) in *D. catenatum*, *P. equestris*, and *A. shenzhenica*, respectively, which were designated as JAZ proteins. The *JAZ* genes in *D. catenatum* and *P. equestris* were almost twice the number of *A. shenzhenica*, which is the original group of orchid [46]. It was observed that the length of the DcJAZ proteins ranged from 123 (LOC110097296) to 401 (LOC110103933) amino acid (aa) residues with an average length of 225 aa. The molecular weight ranged from 13,912.86 to 43,134.7 Da and the pI values varied from 5.50 to 9.34. Subcellular location prediction showed that 10 DcJAZ proteins were localized in the nucleus. Similarly, most JAZ proteins of the other two orchid species were predicted in the nucleus, indicated that JAZ protein mainly functions in the nucleus.

After the removal of redundant gene, 12, 14, and 9 non-redundant MYC genes were identified in *D. catenatum*, *P. equestris*, and *A. shenzhenica*, respectively (Tables 1, S1, and S2). The lengths of 12 DcMYC proteins were ranged from 381 to 662 aa with an average length of 554 aa. The predicted MWs of each DcMYC protein were ranged from 42,301.88 Da to 74,540.42 Da, and the corresponding pIs were changed from 5.18 to 6.92. The pI values of DcJAZs and PeJAZs were much lower than that of AsJAZs, showed that members of the MYC family are also relatively conservative in epiphytic orchids. The predicted subcellular location of DcMYC proteins suggested that all the *D. catenatum* MYCs were localized in the nucleus, consistent with their major roles as transcriptional factors. In addition, all these JA signal pathway genes were named after their *Arabidopsis* homologs.

### Phylogenetic and structural analyses

To identify the classification and evolutionary patterns of COIs, TIFYs and MYCs in *D. catenatum*, we used COI, TIFY, and MYC proteins in *A. thaliana*, *O. sativa*, and its close relative *P. equestris* and *A. shenzhenica* as references for phylogenetic analysis, respectively.

**Fig. 2:**
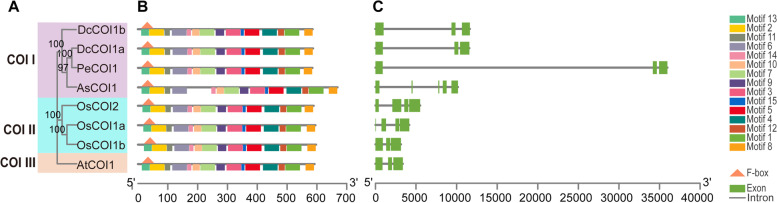
Phylogenetic relationships, architecture of conserved protein domains and motifs, and gene structure in *COI* genes of *D. catenatum*, *P. equestris*, *A. shenzhenica, A. thaliana,* and *O. sativa*. **A** The phylogenetic tree of COI proteins. It was constructed according to the NJ method by MEGA 7.0 with 1000 bootstrap replicates. **B** The conserved domain and motif composition of COI proteins. The Solid triangle with orange colors represented the F-box domain. The Solid boxes with different colors represented different motif, and the legend was given at the right of figure. **C** Exon–intron structure of *COI* genes. Green boxes represents exons and black lines represent introns

Eight COI proteins from the above five species were clustered into three groups, COII-COIIII, and the four protein sequences from three orchid species clustered into the same group (Fig. 2A), revealing that COI protein is highly conserved in evolution among orchid species, a similar phenomenon was also observed in the members of TIFY and MYC family as mentioned later. Conserved domain analysis revealed that all the COI proteins contain typical F-box and 10–14 tandem LRR repeats (Figs. 2B and S1). DcCOI1b, PeCOI1, and OsCOI1a contained 13 LRRs with similar location distribution (Figure S1), indicating that DcCOI1b and PeCOI1 maybe harbor a similar function with OsCOI1a. The *COI* genes contain 2 ~ 4 introns (Fig. 2C), which are extremely long in the three orchid species compared with those of *Arabidopsis* and *Oryza*, as attributed to a large number of repeated sequences in these three orchid genomes [46–48].

**Fig. 3:**
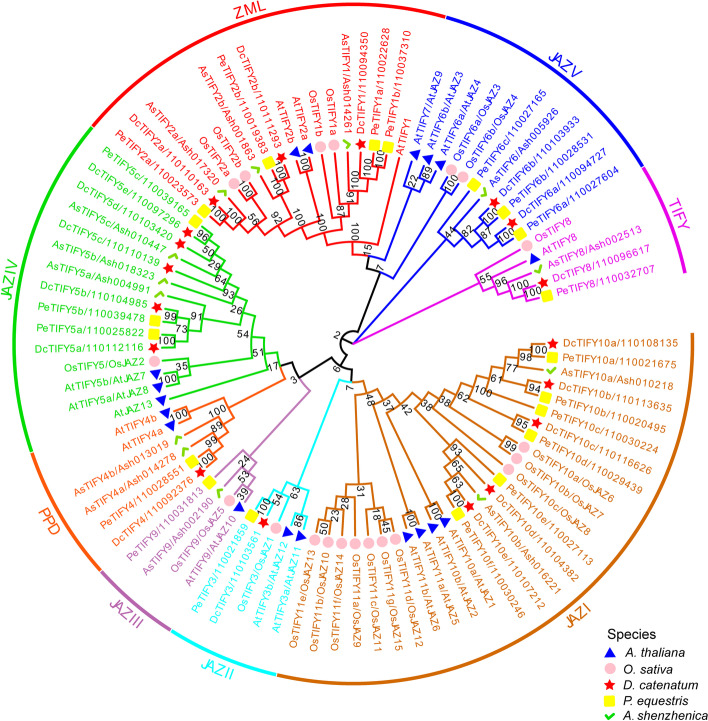
Phylogenetic analysis of 89 TIFY proteins from *D. catenatum*, *P. equestris*, *A. shenzhenica, A. thaliana,* and *O. sativa*. The phylogenetic tree was constructed according to the NJ method by MEGA 7.0 with 1000 bootstrap replicates. DcTIFYs, PeTIFYs, AsTIFYs, AtTIFYs, and OsTIFYs were labeled with red star, yellow square, green hook, blue triangle, pink circle, respectively. The eight groups with different colors represent eight clades

**Fig. 4:**
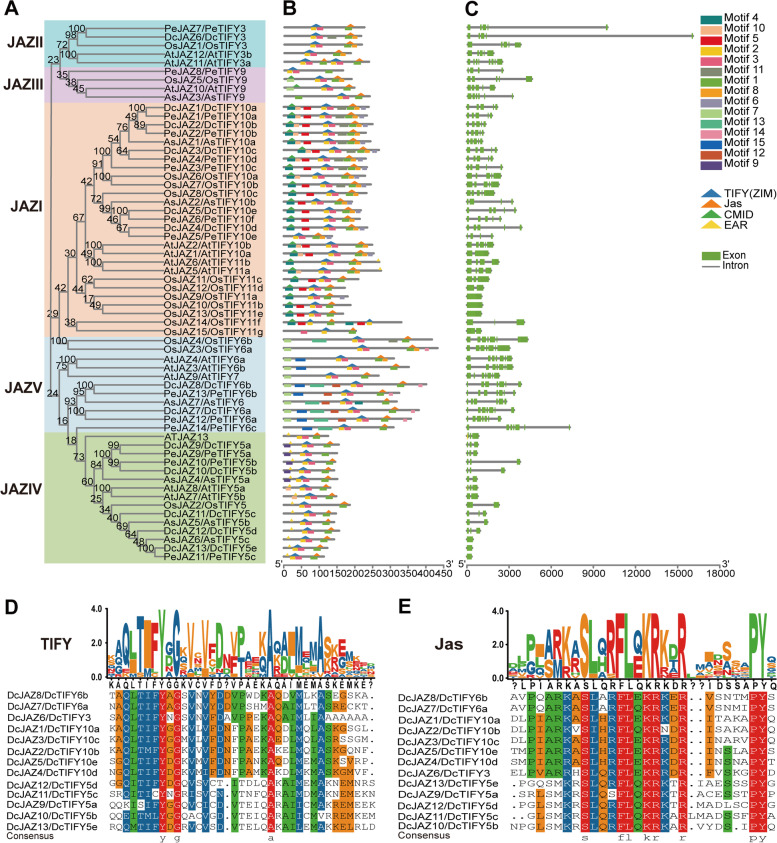
Phylogenetic relationships, architecture of conserved protein domains and motifs, and gene structure in *JAZ* genes of *D. catenatum*, *P. equestris*, *A. shenzhenica, A. thaliana,* and *O. sativa*. **A** The phylogenetic tree of JAZ proteins. Different color boxes represent five different clades. **B** The conserved domain and motif composition of JAZ proteins. The Solid triangle with different colors represented different conserved protein domains (TIFY, Jas, CMID, and EAR). The Solid boxes with different colors represented different motifs, and the legend was given at the right of the figure. **C** Exon–intron structure of *JAZ* genes. Green boxes represent exons and black lines represents introns. Sequence logos of the TIFY (**D**) and Jas (**E**) domains from *D. catenatum* JAZ proteins

As shown in Fig. 3, TIFY proteins of the above five species were classified into eight groups (JAZI, JAZII, JAZIII, JAZIV, JAZV, TIFY, ZML, and PPD). Among them, the classification of JAZ subfamily is consistent with that of *Arabidopsis* [42]. As expected, the homologous genes in three orchid species always cluster together within one branch. DcTIFYs distributed in seven groups except JAZIII, indicating that the JAZIII branch was lost in the divergence from the *D. catenatum* and *P. equestris*. JAZI was the largest group with 5, 6, 2, 10, and 4 genes in *D. catenatum*, *P. equestris*, *A. shenzhenica*, *O. sativa,* and *A. thaliana*, respectively. The high number of JAZI genes in *D. catenatum*, *P. equestris*, and *O. sativa* is consistent with the view that significant expansion of JAZ I group genes in monocots [13], but only two members in *A.* s*henzhenica* indicated that the basal species in the orchid have undergone gene loss during the evolution [46]. Subsequent structural analysis focused on JAZ subfamily (Fig. 4B and C). The genomic sequences of most of these genes were less than 5 kb, but *DcJAZ6* had the longest genomic sequence of approximately 16 kb. The clade JAZV had the longest open reading frame (ORF), whereas JAZIV clade had the shortest ORF. Insights into the phylogeny and gene structures of JAZ genes indicated that proteins that are closely related phylogenetically have similar gene structures. In addition, all of the JAZ genes had symbolic ZIM domain and Jas motif (Fig. 4B, D and E). The sequence logo for the TIFY domain (Fig. 4D), generated using all DcJAZs, showed that the *D. catenatum* TIFY domain was remarkably conserved and very similar to other plant species [22, 49–51]. Besides, the Jas motifs of DcJAZs display the consensus sequence SLX_2_FX_2_KRX_2_RX_5_PY (Fig. 4E), which involved in the interaction with COI and MYCs [52]. Consist with AtJAZs, the JAZIV clade proteins contained an EAR motif that mediates direct interaction with TPL without NINJA (Fig. 4B). In addition, same to AtJAZI and AtJAZIII group in *Arabidopsis*, DcJAZI clade showed an N-terminal CMID sequence (Fig. 4B), however, no CMID was identified in DcJAZ III clade. The motif distribution was provided in Fig. 4B. JAZ genes within the same clade were usually found to share a similar motif composition, which was conspicuous in class JAZIV. Motif analysis showed conserved motif was not necessarily within a conserved functional domain. The functions of most of these conserved motifs remain to be elucidated.

**Fig. 5:**
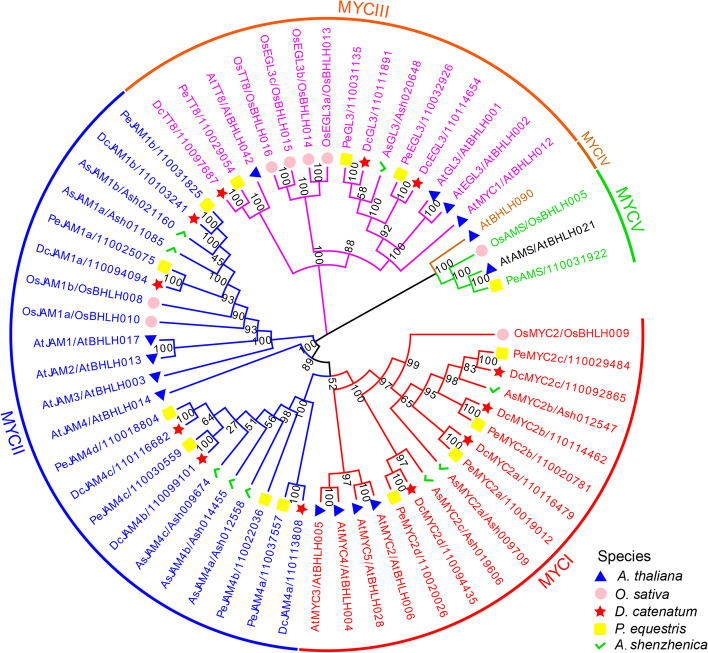
Phylogenetic analysis of 57 MYC proteins from *D. catenatum*, *P. equestris*, *A. shenzhenica, A. thaliana,* and *O. sativa*. The phylogenetic tree was constructed according to the NJ method by MEGA 7.0 with 1000 bootstrap replicates. DcTIFYs, PeTIFYs, AsTIFYs, AtTIFYs, and OsTIFYs were labeled with red star, yellow square, green hook, blue triangle, pink circle, respectively. The five groups with different colors represent five clades

**Fig. 6:**
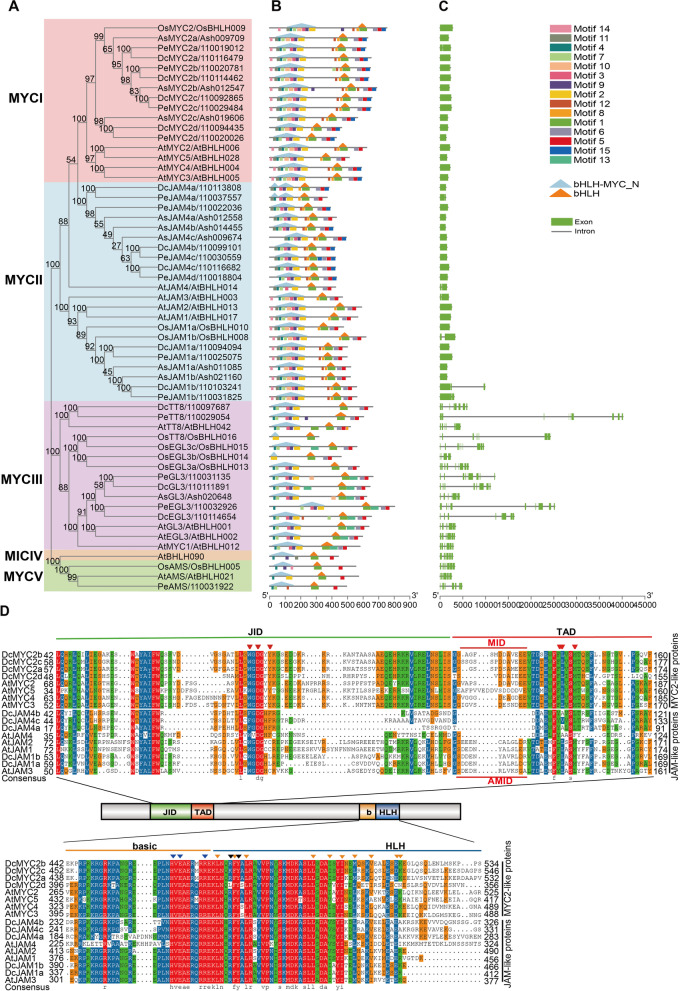
Phylogenetic relationships, architecture of conserved protein domains and motifs, and gene structure in *MYC* genes of *D. catenatum*, *P. equestris*, *A. shenzhenica, A. thaliana,* and *O. sativa*. **A** The phylogenetic tree of MYC proteins. Different color boxes represent five different clades. **B** The conserved domain and motif composition of MYC proteins. The Solid triangle with orange and light blue represented bHLH domain and bHLH-MYC_N domain, respectively. The Solid boxes with different colors represented different motif and the legend was given at the right of figure. **C** Exon–intron structure of *MYC* genes. Green boxes represent exons and black lines represent introns. **D** Sequence alignment of JID, TAD, basic, and HLH domain of DcMYC2a, DcMYC2b, DcMYC2c, DcMYC2d, AtMYC2, AtMYC3, AtMYC4, and AtMYC5 proteins. Sequence alignment was performed using ClustalX. Red triangles represented amino acid residues involved in the *At*MYC3-JAZ interaction. Blue triangles represented residues that are related to specific DNA recognition in MYC2. Orange triangles and black triangles represented residues that are required for MYC2 dimer formation and tetramer formation, respectively

A MYC family phylogenetic tree was generated using 12 DcMYCs, 14 PeMYCs, 9 AsMYCs, 8 OsMYCs, and 14 At MYCs. These MYCs were classified into five subgroups with highly bootstrap values (Fig. 5). The class MYCII was the largest, while the class MYCIV only has one member. Four, 5, and 3 DcMYCs were clustered into MYCI, MYCII, and MYCIII, respectively. However, no DcMYC was detected in MYCIV and MYCV, which may be experienced gene loss in evolution. It was worth noting that, in MYCI group, four MYC2-like proteins in *D. catenatum* were clustered with the other three monocots, while AtMYC2/3/4/5 were clustered in a single branch, indicating that gene replication was carried out after dividing into dicots and monocots, resulting in paralogs (Figs. 5 and 6A). There were five *D. catenatum* JA-ASSOCIATED MYC2-LIKE (JAM) members in MYCII clade, which orthologs in *Arabidopsis* is a negative regulator of JA signaling pathway. MYCII clade members had similar motif distribution and gene structure with MYCI clade members. MYCI and MYCII genes usually consist of only one exon, significantly less than the other three clades, revealing the MYC orthologs were remarkably conserved among different species (Fig. 6C). The intron–exon structure of different DcMYC family members was diverse, while the same subclade genes were similar or same, such as MYC2-like (*DcMYC2a/2b/2c/2d*) and JAM4-like (*DcJAM4a/4b/4c*).

### Tissue-specific expression patterns of core JA signaling pathway genes

To gain insights into the potential roles of core JA pathway genes during development in *D. catenatum*, the published transcriptomic data were investigated in various tissues and organs, including vegetative (leaf, root, green root tip, the white part of root, and stem) and reproductive (flower bud, sepal, labellum, pollinia, and gynostemium) organs.

**Fig. 7:**
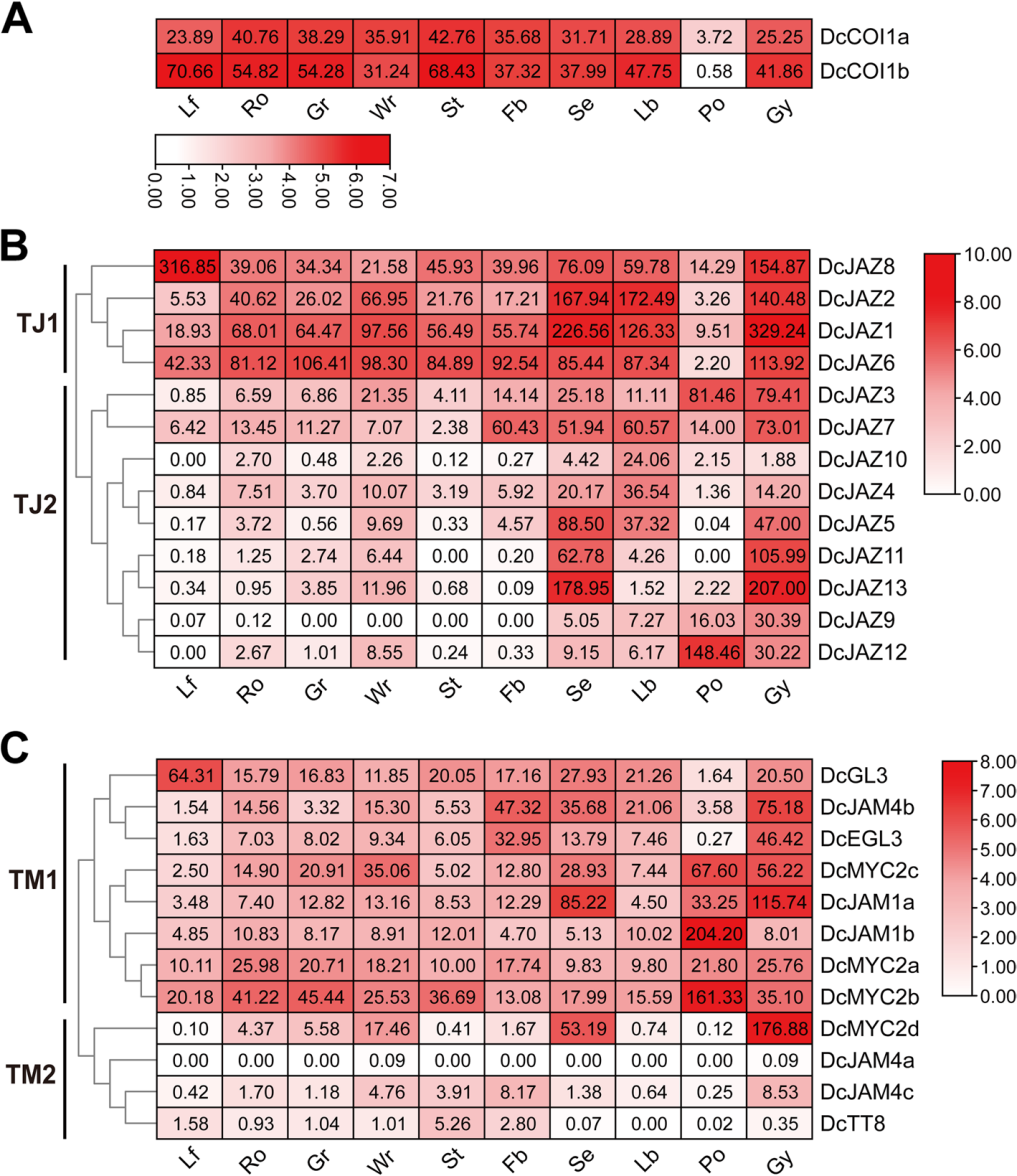
Expression profiles of JA signaling pathway genes in different tissues and organs in *D. catenatum*. **A** Expression patterns of *DcCOI* genes in ten *D. catenatum* tissues and organs. **B** Expression patterns of *DcJAZ* genes in ten *D. catenatum* tissues and organs. **C** Expression patterns of *DcMYC* genes in ten *D. catenatum* tissues and organs. Lf: leaves, Ro: roots, Gr: green root tip, Wr: the white part of roots, St: stems, Fb: flower buds, Se: sepals, Lb: labellum (lip), Po: pollinia, and Gs, gynostemium (column). The log_2_ transformations of the expression values were used to generate the heat map with TBtools software

According to hierarchical clustering (Fig. 7A), *DcCOI1a* and *DcCOI1b* exhibited constitutive expressions with high levels in all tissues and organs except pollinia. Results of JAZ family members indicate that the expression profiles of *DcJAZs* could be split into two groups, TJ1 and TJ2 (Fig. 7B). Members of the TJ1 group (*DcJAZ1*/*2*/*6*/*8*) usually featured high expressions in most tissues. Exceptionaly, all TJ1 genes showed low expressions in pollinia, and *DcJAZ1*and *DcJAZ2* exhibited low expression in leaf. The TJ2 genes (*DcJAZ3*/*4*/*5*/*7/9/10/11/12/13*) witnessed low expression levels in the vegetative organs, while some of these genes showed high transcriptional levels in special reproductive organs, such as *DcJAZ7* in all reproductive organs except pollinia, *DcJAZ3* in pollinia and gynostemium, *DcJAZ12* in pollinia, and *DcJAZ5*/*11*/*13* in sepal and gynostemium. These findings suggested that TJ1 genes might play essential roles in plant growth and development and TJ2 genes displayed prominent functions in flower development. The expression characteristics of MYC family genes in different tissues were quite diverse (Fig. 7C). The transcript abundance of *DcMYCs* could be also divided into two groups, TM1 and TM2. The majority of TM1 genes showed intermediate expressions in most of the detected tissues, whereas several expressed at high levels in specific tissues, such as *DcGL3* in leaf, *DcJAM4b* and *DcEGL3* in gynostemium, *DcMYC2c* in pollinia and gynostemium, *DcJAM1a* in sepal and gynostemium, *DcJAM1b* and *DcMYC2b* in pollinia, suggesting that these genes might play essential roles in specific tissue and organ development. TM2 genes (*DcMYC2d, DcJAM4a, DcJAM4c,* and *DcTT8*) showed low expressions in most tissues. However, *DcMYC2d* are highly expressed in sepal and gynostemium, illustrating it might perform a dominant function during sepal and gynostemium development.

### Transcriptome sequencing

To gain further insights into the role of exogenous MeJA application in the enhanced resistance to *S. delphinii* in *D. catenatum*, a comparative transcriptome analysis was performed. Four leaf samples were obtained at 24 h after inoculated with sterile distilled water (mock) or *S. delphinii* (P1) from plantlets pre-treated with 0 or 1 mM MeJA (CK, P1, JA, and P1 + JA), respectively. All samples used for transcriptome sequencing have three biological replicates (Table S3). Using the HiSeq4000 sequencing platform, 107.83–148.06 million raw reads were generated for each RNA sample. Based on the *D. catenatum* genome, the number of mapped clean reads was 47.31 ~ 69.65 million (Table S4). Comparison of the transcript abundances in the leaves of CK, P1, JA, and P1 + JA, 5255 differentially expressed genes (DEGs) were detected (Table S5). Comparison analysis of the plantlets inoculated with *S. delphinii* pre-treated by MeJA (P1 + JA) and those only MeJA pre-treated (JA), 982 DEGs were identified, with 825 significantly upregulated and 157 significantly downregulated genes. Comparing the control plantlets and MeJA-pretreated plantlets after *S. delphinii* inoculation (P1 vs P1 + JA) uncovered 848 genes significantly up-regulated in the latter (Table S5).

### JA pathway genes significantly responds to *S. delphinii*

**Fig. 8:**
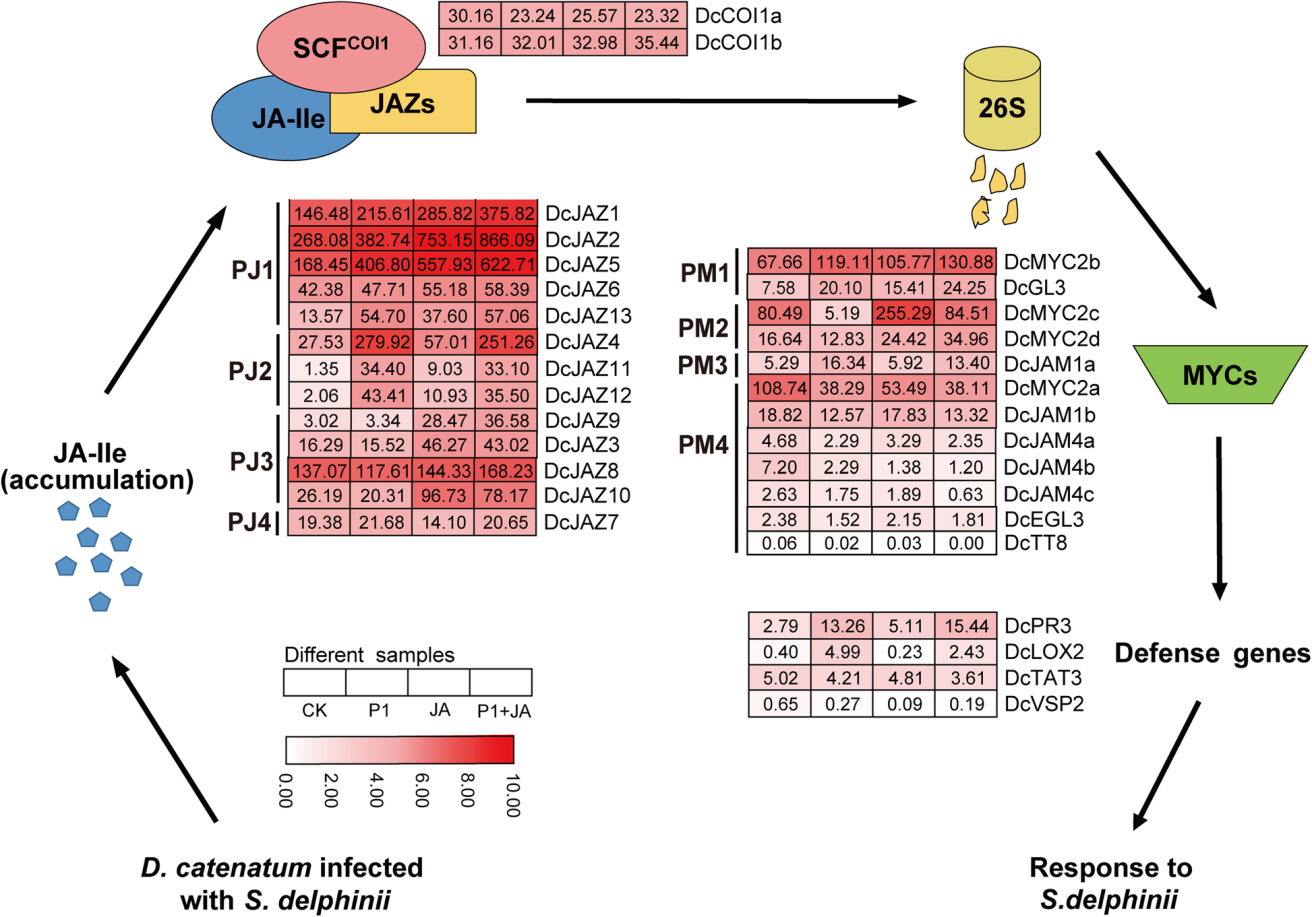
Expression profiles of JA signaling pathway genes response to *S. delphinii* after MeJA pre-treated. Heat map showing expression pattern of *DcCOI*, *DcJAZ*, *DcMYC*, and JA pathway marker genes (*DcPR3, DcLOX2, DcTAT3,* and *DcVSP2*) in leaves under different treatments. CK: control. P1: 24 h post inoculated with *S. delphinii. JA:* pre-treated by MeJA for 4 h and then inoculated with sterile distilled water for 24 h. P1 + JA: pre-treated by MeJA for 4 h and then inoculated with *S. delphinii* for 24 h. The log_2_ transformations of the expression values were used to generate the heat map with TBtools software

Comparative transcriptome changes for genes involved in JA pathway response to pathogen and MeJA were investigated (Table S6). Figure 8 shows the *S. delphinii* (P1) and MeJA-induced expression pattern changes of 31 related genes in this pathway. Gene expression levels were illustrated using a heatmap and estimated by log scale. The transcription levels of the two *DcCOI1* genes did not shown significantly changes after MeJA treated or *S. delphinii* infected (Fig. 8 and Table S6). This result may be attributable to the fact that COI1 as JA receptor is functionally regulated mainly in protein level but not in transcription level. Most of the *DcJAZ* genes were significantly upregulated in three treatment samples (P1, JA, P1 + JA) compared with the control (CK). The expression pattern of these JAZ genes can be divided into four categories (Fig. 8). The PJ1 class genes (*DcJAZ1*/*2*/*5*/*6*/*13*) were significantly induced by MeJA or *S. delphinii*, respectively, and the expression had an additive effect when MeJA and *S. delphinii* were both treated; the PJ2 class genes (*DcJAZ4*/*11*/*12*) were significantly upregulated by MeJA or *S. delphinii* respectively, but it was more significantly response to *S. delphinii* than MeJA. However, there was no evidently expression difference between P1 and P1 + JA, indicating that these genes are responsive to *S. delphinii* but not MeJA; the PJ3 class genes (*DcJAZ3*/*8*/*9*/*10*) were only induced by MeJA, suggesting that these type genes did not respond to *S. delphinii*, and may be involved in the growth and development process related to JA pathway; the fourth type of genes (*DcJAZ*7) was not detected obvious transcriptional changes. The expression of MYC family genes was more complicated, and its expression profiles could also be divided into four types (Fig. 8). The expression patterns of PM1 (*DcMYC2b* and *DcGL3*) and PM3 (*DcJAM1a*) group were consistent with PJ1 and PJ2 classes of *JAZ* genes, respectively. The PM2 (*DcMYC2c*/*2d*) class genes showed that *S. delphinii* inhibited but MeJA significantly induced expression, indicating that this type of genes was a negative regulator in *D. catenatum* response to *S. delphinii*; the PM4 class genes were significantly downregulated in samples P1, JA, and P1 + JA, which was consistent with their role as negative regulators of JA pathway. However, JA-responsive genes, *DcPR3* (LOC110093420) and *DcLOX2* (LOC110106362) showed significantly induced in P1, JA, and P1 + JA samples, but with lower expression levels. *DcTAT3* (LOC110109169) and *DcVSP2* (LOC110107086) exhibited weak induction.

**Fig. 9:**
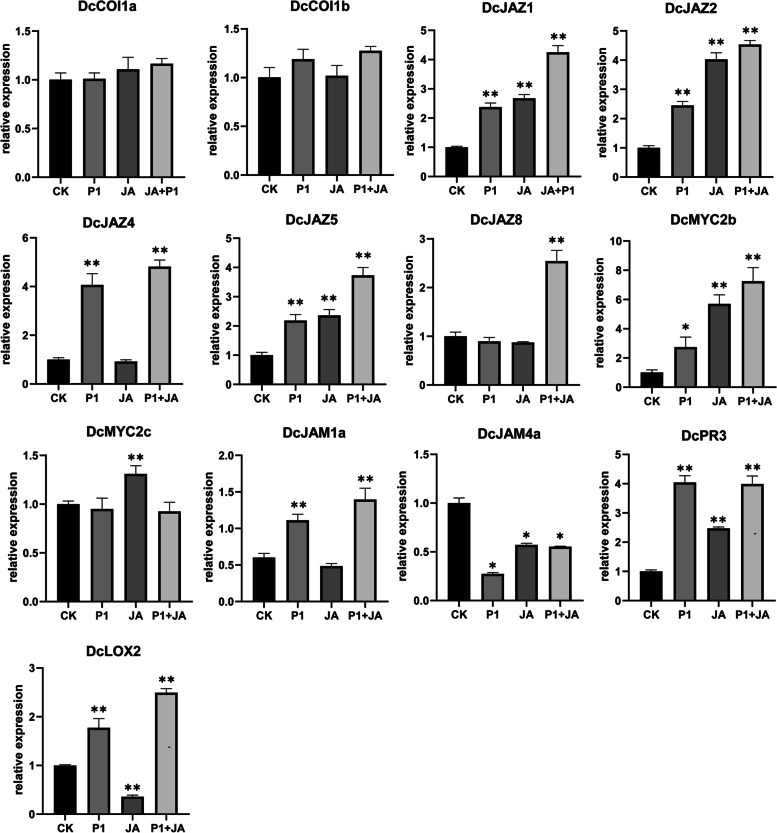
Expression of 11 JA signaling pathway genes response to *S. delphinii* after MeJA-pretreated through RT-qPCR assay. CK: control. P1: 24 h post inoculated with *S. delphinii. JA:* pre-treated by MeJA for 4 h and then inoculated with sterile distilled water for 24 h. P1 + JA: pre-treated by MeJA for 4 h and then inoculated with *S. delphinii* for 24 h.The actin gene of *D. catenatum* was used as an internal control. The error bars indicate SD from three independent experiments. The * and ** show the significant difference at *P* < 0.05 and *P* < 0.01 compared with the CK by Student’s test, respectively

Real-time quantitative PCR of 13 representative genes confirmed the RNA-sequencing data (Fig. 9). *DcCOI1a* and *DcCOI1b* had no markedly expression changes. *DcJAZ1*, *DcJAZ2* and *DcJAZ5* were tightly co-expressed with *DcMYC2b*, with their transcript levels significantly upregulated in P1, JA, and P1 + JA treatment samples. *DcJAM4b* showed negative regulation, while the transcript levels of *DcJAZ4* and *DcJAM1a* were high in P1 and P1 + JA samples but low in JA treated sample. *DcPR3* and *DcLOX2* were significantly induced in three treated samples. *DcMYC2c* induced by JA but not seriously respond to *S. delphinii*, while *MYC2c* significantly downregulated after P1 treatment in transcriptome data. The *DcJAZ8* was significantly upregulated in P1 + JA, which was inconsistent with transcriptome expression data.

## Discussion

Southern Blight is one of the most destructive fungal diseases against *D. catenatum* planting, causing almost no yield [5]. However, an understanding of the mechanism(s) underlying *D. catenatum* resistance to *S. delphinii* has not been reported. After decades of research, JA has been firmly established as a central role in mediating defense response and facilitate the adaptation of plants to a wide range of biotic and abiotic stresses, especially in defenses to necrotrophic pathogens and chewing insects [12, 42]. In this study, *D. catenatum* plantlets pre-treated with MeJA obviously alleviated *S. delphinii* infection symptoms, indicating that the MeJA was useful for enhancing *D. catenatum* resistance to *S. delphinii* (Fig. 1).

The F-box protein COI1 was isolated in 1990s, which is the first milestone in JA signaling pathway [22, 53]. Both JA related pathways are depend on the *COI1* gene. In *Arabidopsis*, a single *COI1* (At2939940) gene was characterized and *coi1* mutant exhibits male sterile, resistant to a coronatine-producing strain of *pseudomonas syringae*, insensitive to MeJA, and sensitive to *B. cinerea* [53, 54]. In the rice genome, three COI homologs were identified [55], i.e., OsCOI1a (Os01g0853400), OsCOI1b (Os05g0449500), and OsCOI2 (Os03g0265500). OsCOI1a and OsCOI1b share over 80% amino acid sequence similarity, while OsCOI2 shows fewer sequence identities (63%) with the two OsCOI1 proteins. Moreover, *OsCOI1* RNAi lines, targeting a sequence conserved in *OsCOI1a* and *OsCOI1b*, show altered plant height, internode length, grain length, and increased susceptibility to herbivores [56, 57]. Here, 2 COI1 genes were identified in *D. catenatum*. Compared with *Arabidopsis*, the number change of COI genes in *D. catenatum* may attribute to undergone two whole-genome duplication (WGD) events (a τ WGD shared by most monocots and a recent WGD specific to the Orchidaceae lineage [46, 58]. The gene structure and motif distribution of the pair of *DcCOI1* genes, *DcCOI1a/1b,* were conserved. Moreover, *DcCOI1a/1b* expressed in all examined tissues with similar expression patterns (Fig. 8), indicating that they might have experienced subfunctionalization [59]. It is intriguing that *DcCOI1a/1b* expressed in pollen were significantly lower than the other tissues. Previous study found that COI performs functions at the protein level and a proper protein level of COI1 is important to maintain distinct JA responses [60].

The plant-specific group JAZ proteins plays a pivotal role in MYC TFs activity change and has emerged as nodes for signal crosstalk in hormone response pathways. *Arabidopsis* genome contains 13 JAZ members that are classified into five groups (I-V) [42]. In the present study, 13 DcJAZ proteins were identified and classify based on the homology with that of Arabidopsis (Fig. 4A and Table 1). However, no DcJAZs were found in JAZIII group. This result is inconsistent with the view that all the five groups exist in all angiosperms [13]. We speculated that group III genes might have been experienced specifically lost during the evolution of *D. catenatum*. The domain architecture of JAZI group proteins (DcJAZ1-5) was more similar to AtJAZ1 and AtJAZ2, containing a CMID domain but no EAR domain [42]. Among them, *DcJAZ1*/2/*5* were simultaneously significantly induced by P1, JA, and both (PJ1 expression mode, Fig. 8), indicating that these three *DcJAZ*s played a critical part in enhancing the resistance to *S. delphinii* in *D. catenatum*. *DcJAZ4* exhibited specific significantly respond to *S. delphinii* but weaker respond to MeJA treatment (PJ2 expression mode, Fig. 8), indicating that *DcJAZ4* acted more as a hub to interact with other pathways (ethylene-JA, salicylic acid (SA)-JA etc.) to enhance the defense responses to pathogens in *D. catenatum*. *DcJAZ3* showed PJ3 expression pattern and was significantly induced by JA instead of *S. delphinii*, speculating that *DcJAZ3* might be involved in JA-mediated growth and development pathways. The single member of JAZII group, *DcJAZ6*, exhibited PJ1 expression pattern but no significant changes detected. Its *Arabidopsis* homologous gene mutant *jaz12-1* shows normal JA responses and performs a redundant function in the JA response. Therefore, *DcJAZ6* might function redundantly with *DcJAZ1*/*2*/*5* in *D. catenatum*. The JAZIV group with five members had the shortest protein length in all the five groups (Fig. 4B and Table 1). Its close *Arabidopsis* homologues AtJAZ7, AtJAZ8, and AtJAZ13 harbors a divergent Jas motif that interacts weakly with COI1 but is able to interact with MYC2, and behaves as a constitutive repressor in the jasmonate pathway [25, 32, 33]. The *DcJAZs* in this group showed three expressing patterns (PJ1, PJ2, and PJ3), meaning they functions in different JA pathways act as inert JAZ proteins. The JAZV group members (DcJAZ7 and DcJAZ8) showed different structures with the other four groups with the longest protein length and the highest number of exons. In *Arabidopsis,* group JAZV proteins includes AtJAZ3, AtJAZ4, and AtJAZ9 [42]. *Atjaz9-1* mutant has normal JA-mediated responses but a specific role in the JA-GA crosstalk, acting functional redundancy in JA responses [33, 61]. The knockout lines *Atjaz4-1* were hypersensitive to JA-induced leaf senescence [62]. Combined with the expression profile of *DcJAZ7* (no obvious change) and *DcJAZ8* (significantly response to MeJA but not *S. delphinii*), suggesting that these two genes mainly participated in JA-mediated growth and development in *D. catenatum*. All in all, further in-depth molecular dissection of *DcJAZ1*, *DcJAZ2*, and *DcJAZ5* will enrich the defense mechanisms of *D. catenatum* response to *S. delphinii.*

MYC2 and its closely related paralogs MYC3, MYC4, and MYC5 orchestrated transcriptional reprogramming, which is a central theme of JA pathway [27, 63]. Although the molecular components (COI1/JAZ/MYC2 complex) of JA signaling pathway in plants are relatively conserved, but MYC2 regulates downstream genes is species-specific [31, 37]. In *Arabidopsis*, MYC2 differentially regulates the expression of two groups of JA-induced genes. MYC2 activates NAC019 further positively regulates wound response genes (first group) but inhibits the Ethylene-Response-Factor1 (ERF1) to further negatively regulates pathogen response genes (second group) [64]. *Arabidopsis myc2/jin1* mutant shows increased resistance to *B. cinerea* and *P. cucumerina*, and sensitivity to insect pests [12, 29]. In tomato, MYC2 positively regulates both of these two group genes by activating JA2L and ERF.C3 transcription factors, respectively, and the necrosis area of the tomato MYC2-RNAi line inoculated with *B. cinerea* was significantly larger than those of the wild type [31]. *Arabidopsis* MYC2 and its tomato homolog have distinct action modes. It might be due to recruiting different partner proteins in transcriptional regulatory activities of pathogen response genes [31, 45]. In this study, we identified 12 MYC members with bHLH domain and bHLH-MYC_N domain and classified them into five groups. MYCI group genes (*DcMYC2a/2b/2c/2d*) had close homology with *AtMYC2/3/4/5*. The domain architecture and gene structure of DcMYC2a, DcMYC2b, and DcMYC2c were similar, but shorter protein lengths with compact domain architecture was found in DcMYC2d, which consistent with that DcMYC2a, DcMYC2b, and DcMYC2c had a more closely phylogenetic genetic relationship. However, the expression patterns of these four duplicated *DcMYC2* genes were dramatic differences. *DcMYC2a* exhibited PM4 pattern with negative regulation under both MeJA and *S. delphinii*. *DcMYC2c* and *DcMYC2d* showed upregulated by JA, but repressed by *S. delphinii*, suggesting these two genes might involve in JA-regulated growth and development pathways. *DcMYC2b* showed PM1 expression pattern and were positively regulated by MeJA and *S. delphinii*, suggesting that MYC2b may act as positive regulator and play a key role in JA-mediated defense to *S. delphinii* stress in *D. catenatum*. We also detected the expression level of MYC2-regulated pathogen defense genes (At*PDF1.2* orthologous gene in *D. catenatum* was not found), *DcPR3*, *DcLOX2*, *DcTAT3*, and *DcVSP2*. Transcriptome results showed that *DcPR3* and *DcLOX2* significantly response to *S. delphinii*, which was validated in qRT-PCR analysis (Figs. 8 and 9). Therefore, we speculated the action mode of *DcMYC2b* was same to tomato *MYC2*. It worth further investigate its direct targets by high-throughput ChIP-seq method or yeast one-hybrid system. In MYCII group, five *JAM* genes, termed the *MYC2-TARGETED BHLH1* (*MTB*) genes in tomato [37], were isolated. In *Arabidopsis*, the bHLH subclade IIId proteins, bHLH17/JAM1, bHLH13/JAM2, bHLH3/JAM3, bHLH14/JAM4, were found to compete with MYC2-like TFs by binding to its target gene promoters [37, 65, 66]. MYC2-like TFs interacts with the JAZ protein through the N-terminal JID domain. Adjacent to JID is the transcriptional activation domain (TAD), which is responsible for the interaction with MED25. MED25 is a subunit of the mediator complex, which recruits general transcription machinery such as RNA polymerase II to achieve the transcription factor activity of MYC and initiate its transcription of target genes. The MID region in the TAD region is necessary for the interaction between MYC2 and MED25. JAMs-like TFs have a normal JID domain and an altered MID domain (AMID) and can interact with JAZ but cannot bind to MED25 [37]. Same to *Arabidopsis*, the MID-corresponding region of DcJAMs had been altered (Fig. 6D). Sequence alignment indicated that the MID was generally conserved in MYC2-like proteins and that the AMID was conserved in JAMs-like proteins in *Arabidopsis* and *D. catenatum*. Notably, there was some sequence divergence in AMID domain between DcJAM4s and the other DcJAMs-like proteins and their functional divergence is worthy to further investigation. Transcriptome analysis revealed that four of this group genes (*DcJAM1b*/*4a*/*4b*/*4c*) were downregulated after *S. delphinii*, MeJA, and both treatment, which was consistent with the role as the negative regulators of JAMs-like TFs. However, *DcJAM1a* showed a significantly improved expression response to *S. delphinii*, suggesting a distinct regulated mechanism.

Based on these observations, we propose a model of *D. catenatum* JA signaling pathway response to *S. delphinii*. Upon *S. delphinii* elicitation, *D. catenatum* elevated JA-Ile levels promote SCF^DcCOI1a/1b^-dependent degradation of DcJAZ1/2/5 repressors, thereby DcMYC2b rapidly and directly regulates the transcription of downstream DcMYC2b-targeted TFs (DcMTFs), which in turn regulate the expression of wounding-responsive (*DcPR3*) or pathogen-responsive genes (*DcLOX2*, Figure S2). Further functional study of the DcMTFs will help to clarify this issue.

## Conclusion

In this study, pre-treated with 1 mM MeJA significantly improved the resistance of *D. catenatum* to *S. delphinii*. Based on this phenotype, we identified 2 COI1, 13 JAZ, and 12 MYC proteins involved in core JA pathway of *D. catenatum*. These core JA pathway members of *D. catenatum* were classified and their evolutionary relationships with *Arabidopsis*, rice, and its closely related orchid species *P. equestris* and *A. shenzhenica*, were evaluated depend on phylogenetic relationship and substrate specificity. The expression profiles of *D. catenatum* core JA pathway genes in different tissues and organs was further analyzed. Comparative transcriptome analysis of the core JA pathway genes in *D. catenatum* planets under MeJA, *S. delphinii,* and combined treatments revealed that *DcJAZ1/2/5* and *DcMYC2b* synergistically promote JA mediated defense response in *D. catenatum*. Therefore, further studies are required to understand how *DcJAZ1/2/5* and *DcMYC2b* is regulated and whether and how it regulates other genes (*DcMTFs*) to control *D. catenatum* responds to *S. delphinii*. Our findings may help establish available avenues for plant breeding strategies aimed at southern blight disease resistance via JA-mediated enhanced plant immunity.

## Methods

### Biological materials

*D. catenatum* clonal cultivar ‘Jingpin xianshi’ (6A2B) is susceptible to *S. delphinii*, which was obtained by subculture from stem explants, placed in a modified 1/2 Murashige and Skoog (MS) medium [67], and cultivated in a growth room with a 12 h light/12 h dark photoperiod, 60% relative humidity, and 25 °C temperatures. The seedlings used for experiments were in same growth status and about 5 cm high, 6 leaves and 3 ~ 5 roots, and the stem diameter was about 0.3 cm.

### MeJA treatment

To study the effects of MeJA on *D. catenatum* plantlets, four-month-old plantlets were spotted with 0.25% ethanol solution (control) or a solution containing 1 mM MeJA (Sigma-Aldrich, 392,707) prepared in 0.25% ethanol solution (1 mL of per plantlet). After MeJA pre-treatment for 4 h, *D. catenatum* plantlets were inoculated with *S. delphinii* (named P1), which was isolated and identified in our laboratory [5].

### Pathogen challenge assays

*S. delphinii* grew on PDA medium at 25 °C for 6 days. Five 5 mm agar disks containing mycelia were collected and cultured in 200 mL PDB medium for 5 days at 25 °C with shaking (180 rpm).

Four-month-old *D. catenatum* plantlets pre-treated by MeJA or 0.25% ethanol solution as indicated above were sprayed with mycelia suspensions (2 mL per plantlet). Control plantlets in each treatment were inoculated with an equal volume of sterile distilled water. *S. delphinii* bioassays were performed at 28 ºC under a photoperiod of 12 h light/12 h dark.

### Disease assessment after *S. delphinii* inoculation

The progress of *S. delphinii* infection was followed for 10 days by observing the development of necrosis in the infected leaves, which can be detected at 24 h post inoculation (hpi), and infection ratings were assigned to the inoculated plantlets: 0, no necrosis; 1, one or two leaves showing a small part of necrosis lesion; 2, one or two leaves showing a large area of necrosis lesion; 3, over three leaves showing necrosis and some leaves dropped off; 4, dead/decayed plant [68]. At least 10 plantlets were inoculated in each experiment. Experiments were repeated at least three times with similar results. The disease index was calculated with the following formula: disease index = ((Σ disease grade × the number of infected plantlets) / (total assessed plantlets × 4)) × 100. Photographs were taken at 60 and 240 hpi.

### Genome-wide identification of core JA signaling pathway genes in some representative orchid species

All the COI, TIFY, MYC sequences including genomic DNAs, coding sequence (CDS), and proteins in *A. thaliana* and *Oryza sativa* were downloaded from the plant genomics resource Phytozome v12 (http://phytozome.jgi.doe.gov/pz/portal.html). *D. catenatum*, *P. equestris* and *A. shenzhenica* genome sequences were retrieved from NCBI Genome (https://www.ncbi.nlm.nih.gov/genome/). To acquire the COI domain-containing sequences in *D. catenatum*, *P. equestris*, and *A. shenzhenica*, the protein sequences of *A. thaliana* [22] and *O. sativa* [69] were used as queries to search homologs against NCBI database using BLASTp tool. The hidden Markov model (HMM) profile of TIFY domain (Pfam accessions: PF06200) was downloaded from the Protein family (Pfam) database (https://pfam.xfam.org/) [70], and used as queries to search for potential TIFY proteins in the *D. catenatum*, *P. equestris* and *A. shenzhenica* protein datasets by using HMMER3.0 [71] with an *E-value* cutoff of 1.0E-05. The MYC family protein was filtered for Pfam database identifiers of the bHLH domain (PF00010) and bHLH-MYC_N domain (PF14215), respectively. All the hits were further confirmed to remove the incomplete and redundant sequences.

The acquired sequences were submitted to ExPASy (https://web.expasy.org/protparam/) to calculate the molecular weights (MW) and theoretical isoelectric points (pI). Subcellular localization were predicted using the WoLF PSORT (https://www.genscript.com/wolf-psort.html).

### Gene structure, domain architecture, conserved motif, and phylogenetic relationship analyses

The conserved motifs were investigated by MEME version 5.3 online tool (http://meme-suite.org/tools/meme). The domain organization was analyzed using the Simple Modular Architecture Research Tool (SMART) and NCBI CD search program. The gene structure, protein domain architecture, and motif composition was visualized using the TBtools [72].

Multiple sequence alignment of COI, TIFY, and MYC proteins was performed using ClustalX [73] with default parameters, respectively. Neighbor-joining (NJ) trees were constructed using MEGA7 [74] with a pairwise deletion option for gaps, p-distance method, and bootstrap test of 1000 replicates. The phylogenetic tree was subsequently visualized with EvolView (https://evolgenius.info/evolview-v2/#login).

### RNA-seq analysis

The leaf samples of *D. catenatum* plantlets were pre-treated with 0 (control) or 1 mM MeJA were harvested for transcriptome sequencing at 24 hpi. Three independent RNA samples (biological replicates) were performed for each treatment. RNA was extracted using the MiniBEST Plant RNA Extraction Kit (TaKaRa, Japan) according to the manufacturer’s instructions. The paired-end sequencing was performed on an Illumina Hiseq4000.

The Illumina RNA-seq data generated from the different tissues were downloaded at the NCBI Sequence Read Archive (SRA) provided by Zhang et al. [46]. SRA data of ten tissues in an individual of wild *D. catenatum*, including leaf (SRR4431601), stem (SRR4431600), root (SRR5722140), green root tip (SRR4431599), white part of the root (SRR4431598), flower bud (SRR4431603), sepal (SRR4431597), labellum (SRR4431602), pollinia (SRR5722145), and gynostemium (SRR4431596), were analyzed in this study.

Reads of above-described samples were aligned to the *D. catenatum* genome [47] using HISAT package [75], which initially remove a portion of the reads based on quality information accompanying each read and then maps the clean reads to the reference genome. StringTie was used to assemble the mapped reads of each sample and estimate the expression levels of all genes by calculating FPKM [76]. The differentially expressed genes were defined with |log_2_ (fold change) |> 1 and with an adjusted *P*-value (*q*-value) < 0.05 by R package [77]. Heatmap was generated using TBtools software [72].

### Quantitative real‑time (RT‑qPCR) analysis

Total RNA was extracted using the MiniBEST Plant RNA Extraction Kit (TaKaRa, Japan). The cDNA was reverse transcribed with the PrimerScript RT Enzyme Mix I kit (TaKaRa, Japan). The primers used for expression analysis were designed by Primer Premier 5 (Table S7). qPCR analysis was performed with SYBR® Premix Ex Taq II (TaKaRa, Japan) on CFX96 Touch™ Real-Time PCR System (BIO-RAD, USA) in three technical replicates each for three independent biological replicates. The *DcACTIN* was used as the internal control gene, which was stably expressed in *D. catenatum* plants and not affected by treatments and genotypes. The relative expression levels were evaluated automatically by the Bio-Rad CFX Manager (version 2.3) with 2^−ΔΔCT^ method [3].

## Supplementary Information

**Additional file 1: Table S1.** Characteristics of core JA signaling pathway genes in *P. equestris*.

**Additional file 2: Table S2.** Characteristics of core JA signaling pathway genes in *A. shenzhenica.*

**Additional file 3: Figure S1.** The leu-rich repeats (LRR) in COI proteins.

**Additional file 4: Table S3.** Summary of transcriptome sequencing data.

**Additional file 5: Table S4.** Summary of mapped reads in transcriptome sequencing.

**Additional file 6: Table S5.** Summary of differentially expressed genes in transcriptome analysis.

**Additional file 7: Table S6.** Expression data of JA signaling pathway genes after MeJA and *S. delphinii* treatment in *D. catenatum*.

**Additional file 8: Figure S2.** The Mode of JA signaling pathway response to *S. delphinii* in *D. catenatum*.

**Additional file 9: Table S7.** Primers used for RT-qPCR.

## Data Availability

The RNA-seq datasets used this article are available in the NCBI Sequence Read Archive (SRA) with BioProject accession number PRJNA732289. The data that support the conclusions are within this article and its additional files. All data and plant materials used in current study are available from the corresponding author on reasonable request.

## Abbreviations

aa: Amino acid
AMID: Altered MID domain
CDS: Coding sequence
ChIP-Seq: Chromatin immunoprecipitation-based sequencing
CK: Control
CMID: N-terminal cryptic MYC-interaction domain
COI1: CORONATINE INSENSITIVE1
CWDEs: Cell wall-degrading enzymes
DcMTFs: DcMYC2b-targeted TFs
DEGs: Differentially expressed genes
EAR: ETHYLENE-RESPONSE FACTOR Amphiphilic Repression
EGL1: ENHANCE R OF GLA BRA3 1
ERF1: Ethylene-Response-Factor1
GL3: GLA BRA3
HMM: Hidden Markov model
JAM: JA-ASSOCIATED MYC2-LIKE
Jas: Jasmonates
JAZ: JASMONATE ZIM-DOMAIN proteins
JID: JAZ-interacting domain
LRRs: Leu-rich repeats
MeJA: Methyl jasmonate
MTB: MYC2-TARGETED BHLH1
MW: Molecular weights
NINJA: NOVEL INTERACTOR OF JAZ
NJ: Neighbor-joining
ORF: Open reading frame
Pfam: Protein family database
pI: Theoretical isoelectric points
RT-qPCR: Real-time quantitative polymerase chain reaction
SA: Salicylic acid
SMART: Simple Modular Architecture Research Tool
SRA: Sequence Read Archive
TAD: Transcriptional activation domain
TPL: TOPLESS
TT8: TRANSPARENT TE STA 8
WGD: Whole-genome duplication
