# Supplementary material for: JA signal-mediated immunity of Dendrobium catenatum to necrotrophic Southern Blight pathogen
Source: BMC Plant Biol. 2021 Aug 6;21:360. doi: 10.1186/s12870-021-03134-y (PMC8344041; doi:10.1186/s12870-021-03134-y)
Supplement: Supplementary file 1 — Additional file 1: Table S1. Characteristics of core JA signaling pathway genes in P. equestris. [file 12870_2021_3134_MOESM1_ESM.docx]

Table S1 Characteristics of core JA signaling pathway genes in *P. equestris*

| Gene Family | No. | Name | Synonym | Gene ID | Clade | Exon Number | Protein(aa) | Isoform | pI | MW(Da) | Localization Predicted |
| --- | --- | --- | --- | --- | --- | --- | --- | --- | --- | --- | --- |
| COI Family | 1 | PeCOI1 |  | LOC110018270 |  | 3 | 585 |  | 6.31 | 66328.51 | Cytoplasmic |
| TIFY Family | 1 | PeTIFY1a | PeZML1 | LOC110022628 | ZML | 8 | 295 | 2 | 8.76 | 32071.23 | Nuclear |
|  | 2 | PeTIFY1b | PeZML2 | LOC110037310 | ZML | 4 | 152 | 0 | 5.07 | 16694.91 | Nuclear |
|  | 3 | PeTIFY2a | PeZML3 | LOC110023573 | ZML | 8 | 261 | 2 | 8.59 | 27995.48 | Nuclear |
|  | 4 | PeTIFY2b | PeZML4 | LOC110019383 | ZML | 8 | 325 | 0 | 5.78 | 35199.28 | Nuclear |
|  | 5 | PeTIFY3 | PeJAZ7 | LOC110021859 | JAZII | 5 | 225 | 0 | 5.41 | 24007.08 | Nuclear |
|  | 6 | PeTIFY4 | PePPD | LOC110028551 | PPD | 10 | 357 | 0 | 9.6 | 38804.15 | Nuclear |
|  | 7 | PeTIFY5a | PeJAZ9 | LOC110025822 | JAZIV | 3 | 150 | 0 | 6.58 | 16380.39 | Nuclear |
|  | 8 | PeTIFY5b | PeJAZ10 | LOC110039478 | JAZIV | 3 | 145 | 3 | 8.68 | 16122.71 | Nuclear |
|  | 9 | PeTIFY5c | PeJAZ11 | LOC110039165 | JAZIV | 1 | 113 | 0 | 9.72 | 12706.68 | Nuclear |
|  | 10 | PeTIFY6a | PeJAZ12 | LOC110027604 | JAZV | 7 | 358 | 2 | 8.63 | 38744.85 | Nuclear |
|  | 11 | PeTIFY6b | PeJAZ13 | LOC110028531 | JAZV | 7 | 318 | 0 | 9.21 | 34112.76 | Nuclear |
|  | 12 | PeTIFY6c | PeJAZ14 | LOC110027165 | JAZV | 7 | 309 | 4 | 9.53 | 34088.71 | Nuclear |
|  | 13 | PeTIFY8 | PeTIFY8 | LOC110032707 | TIFY | 5 | 416 | 2 | 8.6 | 43967.31 | Nuclear |
|  | 14 | PeTIFY9 | PeJAZ8 | LOC110031813 | JAZIII | 4 | 165 | 0 | 10.14 | 18044.96 | Chloroplast |
|  | 15 | PeTIFY10a | PeJAZ1 | LOC110021675 | JAZI | 5 | 234 | 0 | 8.92 | 25402.14 | Nuclear |
|  | 16 | PeTIFY10b | PeJAZ2 | LOC110020495 | JAZI | 5 | 231 | 0 | 8.91 | 24890.21 | Chloroplast |
|  | 17 | PeTIFY10c | PeJAZ3 | LOC110030224 | JAZI | 5 | 234 | 2 | 6.92 | 25824.26 | Chloroplast |
|  | 18 | PeTIFY10d | PeJAZ4 | LOC110029439 | JAZI | 5 | 231 | 2 | 8.81 | 25231.71 | Nuclear |
|  | 19 | PeTIFY10e | PeJAZ5 | LOC110027113 | JAZI | 4 | 136 | 2 | 9.65 | 15245.72 | Nuclear |
|  | 20 | PeTIFY10f | PeJAZ6 | LOC110030246 | JAZI | 5 | 214 | 3 | 6.45 | 24263.63 | Nuclear |
| MYC Family | 1 | PeMYC2a |  | LOC110019012 | MYCI | 2 | 618 | 0 | 5.28 | 68088.8 | Nuclear |
|  | 2 | PeMYC2b |  | LOC110020781 | MYCI | 2 | 647 | 0 | 5.44 | 70945.71 | Nuclear |
|  | 3 | PeMYC2c |  | LOC110029484 | MYCI | 1 | 651 | 0 | 5.56 | 71500.41 | Nuclear |
|  | 4 | PeMYC2d |  | LOC110020026 | MYCI | 1 | 428 | 0 | 9.13 | 47625.76 | Chloroplast |
|  | 5 | PeJAM4a |  | LOC110037557 | MYCII | 2 | 369 | 0 | 5.98 | 40542.61 | Nuclear |
|  | 6 | PeJAM4b |  | LOC110022036 | MYCII | 1 | 390 | 0 | 6.11 | 43206.79 | Nuclear |
|  | 7 | PeJAM4c |  | LOC110030559 | MYCII | 1 | 422 | 0 | 6.29 | 45848.84 | Nuclear |
|  | 8 | PeJAM4d |  | LOC110018804 | MYCII | 1 | 429 | 0 | 5.65 | 46744.81 | Cytoplasmic |
|  | 9 | PeJAM1a |  | LOC110025075 | MYCII | 1 | 497 | 0 | 6.01 | 55364.89 | Nuclear |
|  | 10 | PeJAM1b |  | LOC110031825 | MYCII | 2 | 560 | 0 | 6.24 | 61824.16 | Nuclear |
|  | 11 | PeTT8 |  | LOC110029054 | MYCIII | 9 | 603 | 2 | 5.69 | 68476.6 | Nuclear |
|  | 12 | PeEGL3 |  | LOC110032926 | MYCIII | 12 | 802 | 0 | 5.88 | 91245.05 | Nuclear |
|  | 13 | PeGL3 |  | LOC110031135 | MYCIII | 10 | 663 | 2 | 7.55 | 75066.7 | Nuclear |
|  | 14 | PeAMS |  | LOC110031922 | MYCV | 9 | 494 | 2 | 4.98 | 55599.31 | Nuclear |
