# Supplementary material for: JA signal-mediated immunity of Dendrobium catenatum to necrotrophic Southern Blight pathogen
Source: BMC Plant Biol. 2021 Aug 6;21:360. doi: 10.1186/s12870-021-03134-y (PMC8344041; doi:10.1186/s12870-021-03134-y)
Supplement: Supplementary file 2 — Additional file 2: Table S2. Characteristics of core JA signaling pathway genes in A. shenzhenica. [file 12870_2021_3134_MOESM2_ESM.docx]

Table S2 Characteristics of core JA signaling pathway genes in *A. shenzhenica*

| Gene Family | No. | Name | Synonym | Gene ID | Clade | Exon Number | Protein(aa) | Isoform | pI | MW(Da) | Localization Predicted |
| --- | --- | --- | --- | --- | --- | --- | --- | --- | --- | --- | --- |
| COI Family | 1 | AsCOI1 |  | Ash007918 |  | 5 | 669 |  | 8.11 | 76380.33 | Chloroplast |
| TIFY Family | 1 | AsTIFY1 | AsZML1 | Ash014261 | ZML | 8 | 307 | 0 | 5.92 | 33297.47 | Nuclear |
|  | 2 | AsTIFY2a | AsZML2 | Ash017320 | ZML | 7 | 265 | 0 | 6.71 | 28403.9 | Nuclear |
|  | 3 | AsTIFY2b | AsZML3 | Ash001863 | ZML | 7 | 291 | 0 | 5.86 | 31163.77 | Nuclear |
|  | 4 | AsTIFY4a | AsPPD1 | Ash014278 | PPD | 7 | 373 | 0 | 8.1 | 40716.48 | Nuclear |
|  | 5 | AsTIFY4b | AsPPD2 | Ash013019 | PPD | 9 | 368 | 0 | 9.82 | 40577.33 | Nuclear |
|  | 6 | AsTIFY5a | AsJAZ4 | Ash004991 | JAZIV | 2 | 151 | 0 | 9.41 | 16437.56 | Nuclear |
|  | 7 | AsTIFY5b | AsJAZ5 | Ash018323 | JAZIV | 2 | 142 | 0 | 9.71 | 16191.37 | Nuclear |
|  | 8 | AsTIFY5c | AsJAZ6 | Ash010447 | JAZIV | 1 | 142 | 0 | 9.33 | 15942.91 | Nuclear |
|  | 9 | AsTIFY6 | AsJAZ7 | Ash005926 | JAZV | 6 | 318 | 0 | 7.09 | 34681.7 | Nuclear |
|  | 10 | AsTIFY8 | AsTIFY8 | Ash002513 | TIFY | 6 | 414 | 0 | 6.76 | 44305.67 | Nuclear |
|  | 11 | AsTIFY9 | AsJAZ3 | Ash002190 | JAZIII | 6 | 240 | 0 | 10.51 | 26320.78 | Chloroplast |
|  | 12 | AsTIFY10a | AsJAZ1 | Ash010218 | JAZI | 1 | 244 | 0 | 9.45 | 25748.28 | Nuclear |
|  | 13 | AsTIFY10b | AsJAZ2 | Ash016221 | JAZI | 4 | 192 | 0 | 8.69 | 21407.26 | Chloroplast |
| MYC Family | 1 | AsMYC2a |  | Ash009709 | MYCI | 1 | 630 | 0 | 5.7 | 69043.72 | Nuclear |
|  | 2 | AsMYC2b |  | Ash012547 | MYCI | 1 | 687 | 0 | 5.51 | 74505.34 | Nuclear |
|  | 3 | AsMYC2c |  | Ash019606 | MYCI | 1 | 564 | 0 | 5.27 | 60097.35 | Nuclear |
|  | 4 | AsJAM1a |  | Ash011085 | MYCII | 1 | 520 | 0 | 6.06 | 56921.55 | Nuclear |
|  | 5 | AsJAM1b |  | Ash021160 | MYCII | 1 | 518 | 0 | 6.09 | 56685.39 | Nuclear |
|  | 6 | AsJAM4a |  | Ash012558 | MYCII | 1 | 428 | 0 | 7.24 | 46002.93 | Cytoplasmic |
|  | 7 | AsJAM4b |  | Ash014455 | MYCII | 1 | 408 | 0 | 5.87 | 44216.01 | Nuclear |
|  | 8 | AsJAM4c |  | Ash009674 | MYCII | 1 | 492 | 0 | 6.29 | 50429.27 | Chloroplast |
|  | 9 | AsGL3 |  | Ash020648 | MYCIII | 6 | 623 | 0 | 5.24 | 70921.2 | Nuclear |
