# Supplementary figures and images for: JA signal-mediated immunity of Dendrobium catenatum to necrotrophic Southern Blight pathogen
Source: BMC Plant Biol. 2021 Aug 6;21:360. doi: 10.1186/s12870-021-03134-y (PMC8344041; doi:10.1186/s12870-021-03134-y)

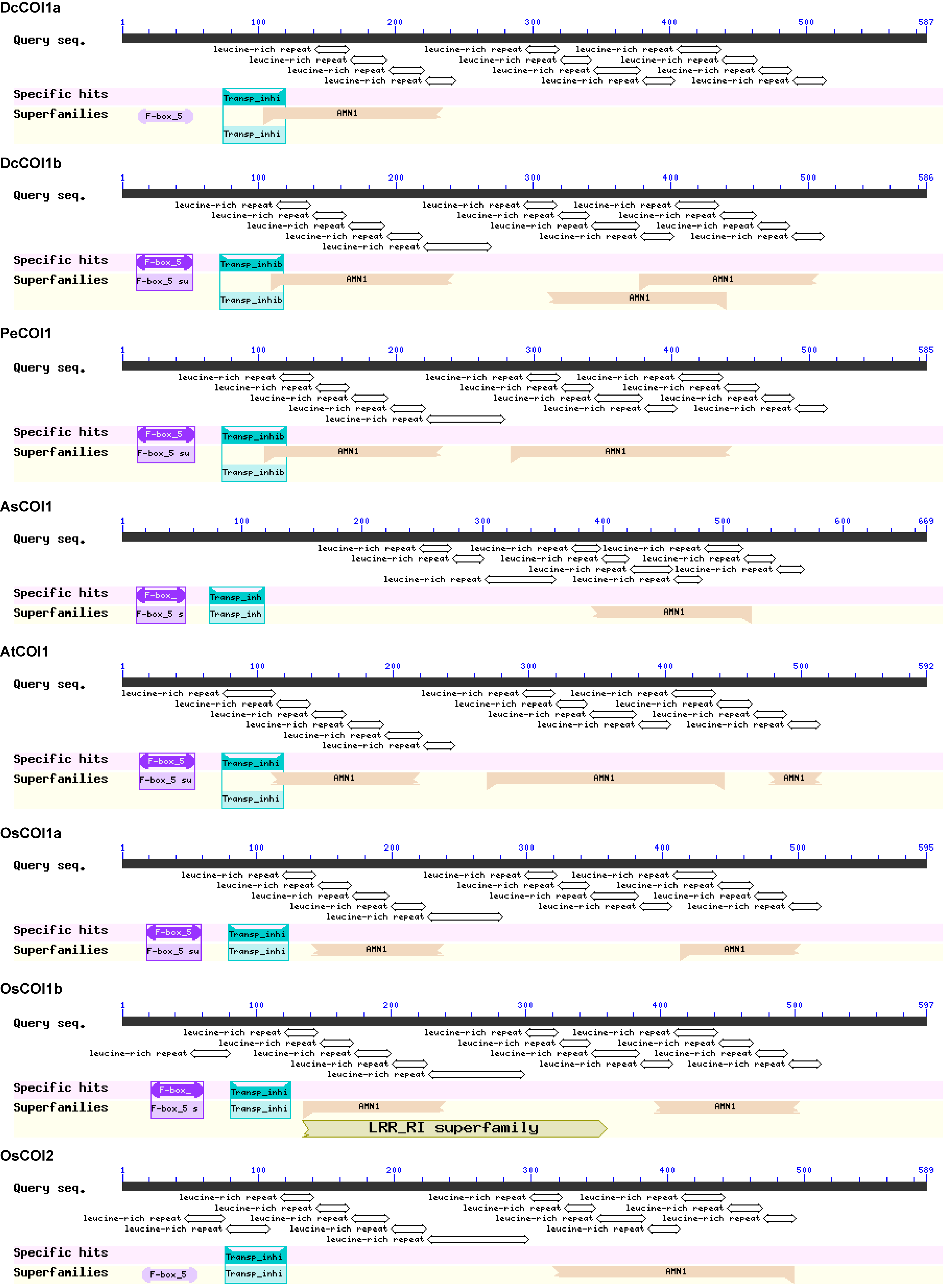

Supplement: Supplementary file 3 — Additional file 3: Figure S1. The leu-rich repeats (LRR) in COI proteins. [file 12870_2021_3134_MOESM3_ESM.tif]

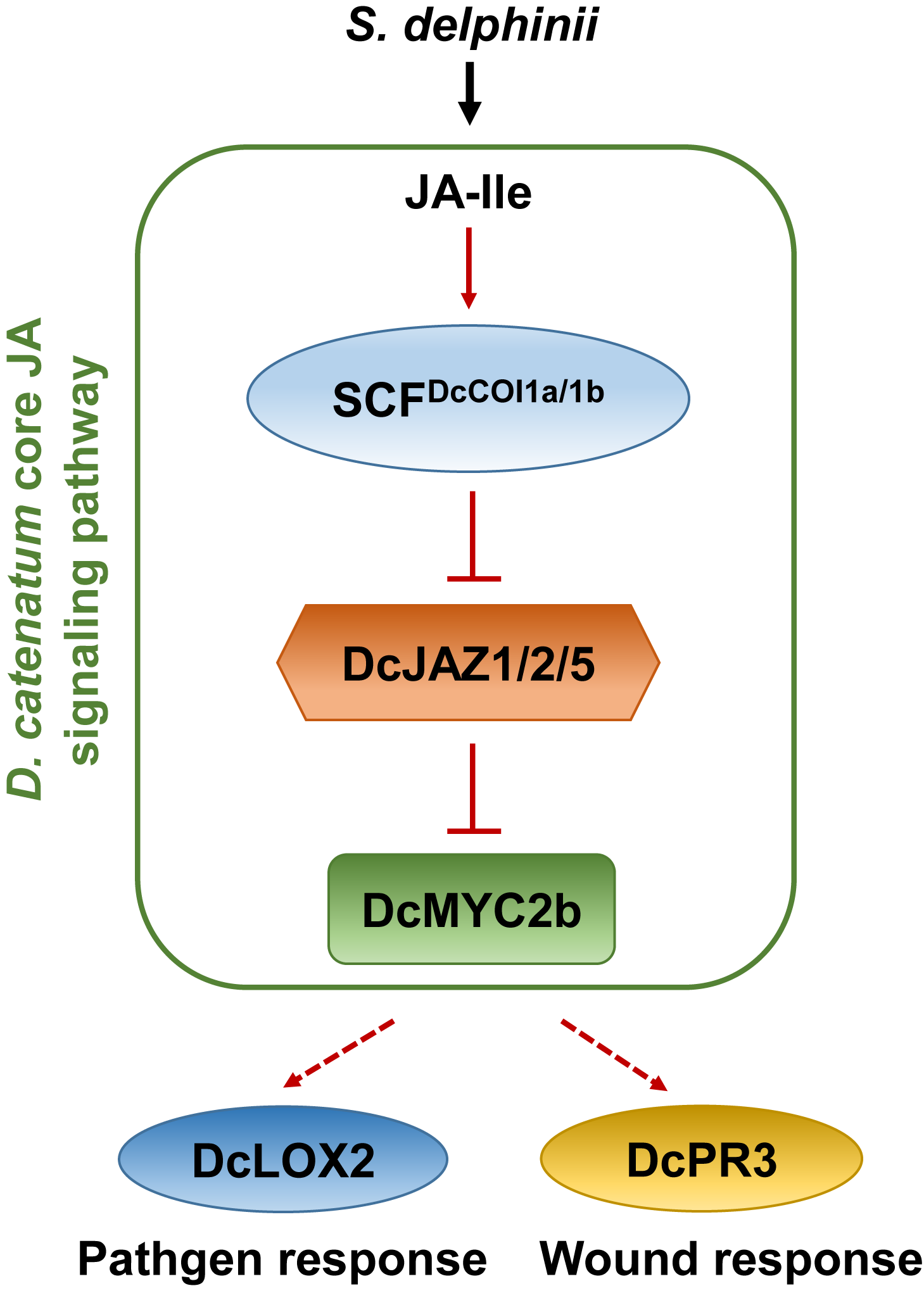

Supplement: Supplementary file 8 — Additional file 8: Figure S2. The Mode of JA signaling pathway response to S. delphinii in D. catenatum. [file 12870_2021_3134_MOESM8_ESM.tif]
